# Unusual surface and edge morphologies, *sp*^2^ to *sp*^3^ hybridized transformation and electronic damage after Ar^+^ ion irradiation of few-layer graphene surfaces

**DOI:** 10.1186/1556-276X-7-466

**Published:** 2012-08-19

**Authors:** Salim Hamood Al-Harthi, Mohammed Elzain, Muataz Al-Barwani, Amal Kora'a, Thomas Hysen, Myo Tay Zar Myint, Maliemadom Ramaswamy Anantharaman

**Affiliations:** 1 Physics Department, College of Science, Sultan Qaboos University, P.O. Box 36, Al Khoud, Sultan Qaboos, Muscat, 123, Oman; 2 Department of Physics, Cochin University of Science and Technology, Cochin-22, Kochi, Kerala, 682 022, India; 3 Center of Excellence in Nanotechnology, Asian Institute of Technology, P.O. Box 4, Klong Luang, Pathumthani, 12120, Thailand

**Keywords:** Few layer graphene, Argon sputtering, Electronic damage, Edge reconstructions

## Abstract

Roughness and defects induced on few-layer graphene (FLG) irradiated by Ar^+^ ions at different energies were investigated using X-ray photoemission spectroscopy (XPS) and atomic force microscopy techniques. The results provide direct experimental evidence of ripple formation, *sp*^2^ to *sp*^3^ hybridized carbon transformation, electronic damage, Ar^+^ implantation, unusual defects and edge reconstructions in FLG, which depend on the irradiation energy. In addition, shadowing effects similar to those found in oblique-angle growth of thin films were seen. Reliable quantification of the transition from the *sp*^2^-bonding to *sp*^3^-hybridized state as a result of Ar^+^ ion irradiation is achieved from the deconvolution of the XPS C (1*s*) peak. Although the ion irradiation effect is demonstrated through the shape of the derivative of the Auger transition C KVV spectra, we show that the *D* parameter values obtained from these spectra which are normally used in the literature fail to account for the *sp*^2^ to *sp*^3^ hybridization transition. In contrast to what is known, it is revealed that using ion irradiation at large FLG sample tilt angles can lead to edge reconstructions. Furthermore, FLG irradiation by low energy of 0.25 keV can be a plausible way of peeling graphene layers without the need of Joule heating reported previously.

## Background

Ion irradiation of materials subjected to different ion energies, ion doses and irradiation geometries such as angle of incidence of the beam, sample tilt angle and sample rotation has been widely studied [1,2]. In addition, most of the underlying ion-matter interaction mechanisms, cascade collisions and irradiation-induced defects have been theoretically explained by Monte Carlo and classical molecular dynamic simulations [3] and density functional theory total energy calculations [4]. However, the discovery of the 2-D crystals such as graphene [5] has introduced new challenges which need new insights. An approach employing transport of ions in matter (TRIM) simulations which is successfully used for bulk material analysis was found not necessarily to work for 2-D crystals [6]. This is due to the fact that in TRIM calculations, the sample is treated as an amorphous matrix with a homogenous mass density neglecting the atomic structure that reflect the nature of atomically flat targets such as graphene. However, a code based on analytical potential molecular dynamic simulations with much more accurate capabilities than TRIM has been developed to account for amorphizations, defects, and single, double and complex vacancies in graphene under ion irradiation as functions of angle of incidence and ion energy [7].

Experimentally, Lopez et al. [8] in their study of a single-layer graphene grown on a SiO_2_/Si substrate exposed to 30 keV Ga^+^ ion irradiation pointed out the requirements for developing a more thorough understanding of graphene's ability to withstand prolonged ion irradiation. These include: (1) the absence of cascade collisions due to the 2-D graphene nature, (2) the possibility of C atoms not to be displaced from the lattice as the ion energy is completely transferred to the substrate underneath, (3) graphene open structure which facilitates the ion channeling, hence limiting the number of ion/target collisions and ejected carbon atoms, and (4) implanted ions which might cause a net positive charge to build up in graphene and electrostatically repel the subsequent incoming incident ions. In addition to the above issues, the importance of this study helps to understand the damage production mechanisms and types of defects created by the energetic ions in the sample for the efficient use of ion beams and optimization of the graphene cutting process [7]. Furthermore, this study suggests the possibility of using graphene membranes in ion beam analysis [9], elimination of surface contaminants from *ex situ* prepared graphene layers [10] and graphene defect-based applications. Although, un-optimized ion irradiation is expected to breakdown the graphene 2-D network, destroy its *sp*^2^ bonding configurations and affect the graphene carrier mobility, this study shade light on defects associated with this process which are equally important. In this respect, applications and mechanisms based on defects in graphene such ferromagnetism [11], creation of metallic wires [12], porous graphene for DNA detection [13], atmospheric pollutant filtration [14] and graphene hydrophobicity enhancement can be realized and understood.

Here, we have employed low energy Ar^+^ with energies from 0.25 to 5 keV at incidence angle of 45° and sample tilt angle of 66° to study the irradiation effects in FLG. In addition to defects found on flat irradiated areas, we show that FLG edge defects associated with *sp*^2^ to *sp*^3^ hybridized carbon transformation are most typical in the low energy range and irradiation geometry used. In addition, we show that the reliable quantification of the *sp*^2^/*sp*^3^ hybridized state ratio as a function of irradiation energy can be achieved from the deconvolution of the XPS C 1*s* envelope. This ratio, together with newly developed features in the Auger transition C KVV spectra and π − π* transition behavior as a function of irradiation energy provide insights on the FLG structural and electronic damage. Furthermore, we point out the possibility of a combined effect of ion implantation and ion reflection to be responsible for the low rate of sputtering of FLG.

## Methods

FLG samples were obtained by peeling layers from highly oriented pyrolytic graphite (HOPG ZYA) using adhesive tape. They were then fixed on steel substrates by double sided stick carbon tape. These substrates were then subjected to Ar^+^ ion irradiation for 30 min for energies ranging from 0.25 to 5 keV at incidence angle of 45° and sample tilt angle of 66° in the ultra high vacuum conditions.

XPS measurements were carried out using an Omicron Nanotechnology XPS system (Omicron NanoTechnology GmbH, Taunusstein, Germany) using a monochromatic Al Kα radiation (hν = 1,486.6 eV). The source voltage and emission current were 15 kV and 20 mA, respectively. The base pressure at which the scans were done was 10^−10^ mbar. The chemical composition of the sample was extracted from the wide scan using CASA XPS software (Fairley, N. *CASA XPS*, version 2.0; CASA Software Ltd., Devon, U.K.). Short scans were recorded at pass energy of 20 eV. In order to avoid charging effect during the scans, an electron gun flooding was used for charge compensation.

The nanoscale images presented were performed using Nanoscope V atomic force microscope (AFM) obtained in tapping mode using ultra high resolution cantilevers made of tungsten having radius of less than 1 nm and force constant of 46 N/m. During the imaging, both the scan rate and the imaging resolution were set at 0.5 Hz and 512 × 512 pixels, respectively.

## Results and discussion

**Figure 1:**
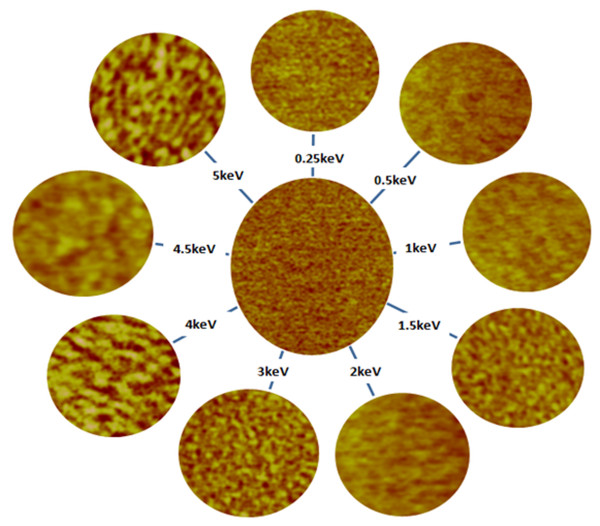
**Sequence of FLG AFM height images as a function Ar**^**+**^**irradiation energy 0.25 to 5 keV.** The image in the middle was obtained from the un-irradiated FLG sample.

**Figure 2:**
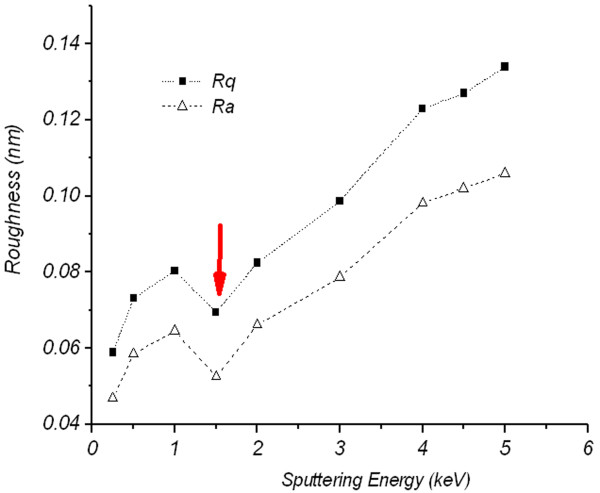
***R***_**a**_**and*R***_**q**_**for FLG as a function of Ar**^**+**^**ion irradiation energy.** Ranging from 0.25 to 5 keV.

Figure 1 represents AFM images for un-irradiated (central image) and irradiated FLG samples at different primary Ar^+^ ion energies ranging from 0.25 to 5 keV. At first glance, roughness and well-known ripple structures of varying sizes and wavelengths [15] are visible after ion beam exposure. Careful analysis of the roughness parameters, mean roughness (*R*_a_) and RMS roughness (*R*_q_) reveals that the roughness is not monotonically increasing as a function of irradiation energy; instead, a reduction in the roughness is found at 1.5 keV as indicated in Figure 2 - a sign of sputtering completion of FLG first layer at this irradiation energy.

**Figure 3:**
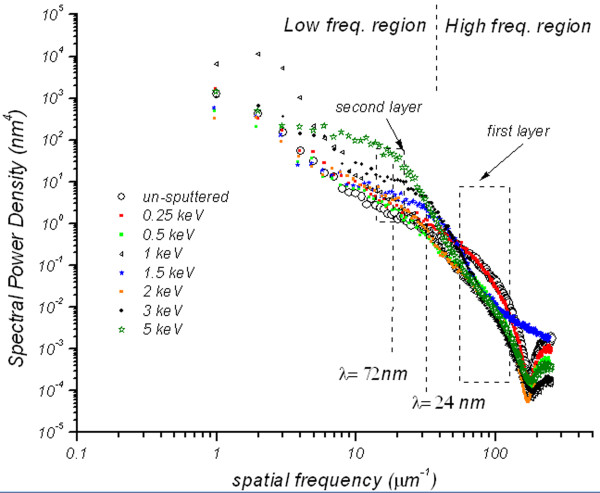
**Two-dimensional PSD spectra calculated from 1 × 1 μm AFM images (energies: 0.25 to 5 keV).** Obtained after irradiation of the samples at different energies from 0.25 to 5 keV. The spectrum for the un-irradiated sample is also plotted for comparison.

The evolution of the large scale morphology was probed by two-dimensional power spectral density (PSD) analysis [16,17]. PSD function provides a representation of the amplitude of a surface's roughness as a function of the spatial frequency. As shown in Figure 3, all PSD functions show a linear dependence at high spatial frequency (*f*) with ripple wavelength (*λ*) of =24 nm. After irradiation at 0.25 keV, the PSD spectrum superimposes to that of the un-irradiated sample, indicating negligible damage of the FLG surface. Interestingly, a decrease in the graphene PSD intensity is observed at energies higher than 0.25 keV for the spatial frequencies between 60 and 120 μm^−1^ (i.e., the area enclosed by a rectangle and labeled as first layer).

**Figure 4:**
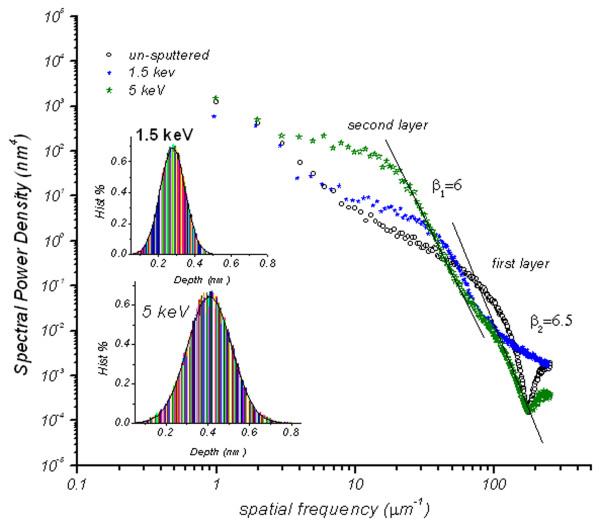
**Two-dimensional PSD spectra calculated from 1 × 1 μm AFM images (energies: 1.5 and 5 keV).** Obtained after irradiation of the samples at 1.5 and 5 keV. The spectrum for the un-irradiated sample is also plotted for comparison. Inset shows the calculated depth distribution after irradiation of samples at 1.5 and 5 keV.

This suggests strong suppression, smoothing and lateral coarsening of ripple structures (i.e., decrease in PSD intensity, hence roughness amplitude) at these energies. At low frequency regime, the spectra show a switching behavior where the PSD intensities are seen to be higher than that of un-irradiated and increase by a factor of 10 and 100 for the 1.5 and 5 keV irradiations, respectively. This increase is a sign of large amplitude ripple formation with long periodicity of *λ* = 72 nm that resulted from sputtering of both the first and the second graphene layers. It should be noted that the irradiation by 1.5 and 5 keV will only affect the first and the second FLG layers; hence, the assignment of ‘second layer’ and ‘first layer’ is used in Figure 3. This is supported by a comparison of depth data obtained from 1 × 1 μm AFM images after irradiation of the samples at 1.5 and 5 keV. The depth is based on the accumulation of data within specified area of the image, application of a Gaussian low-pass filter to the data to remove noise, and then obtaining depth comparisons between dominant features in a consistent, statistical manner [18]. The results of this method are depicted in the inset of Figure 4. Considering 0.34 nm as the thickness of individual atomic planes in graphite [19], the depth distribution seen in the histograms confirms the sputtering of one and two layers after 1.5 and 5 keV irradiations, respectively.

In order to further shed light on the morphology of the two layers after the 5-keV ion irradiation, the roughness exponent α has been determined from the 5-keV PSD spectrum in the high frequency region. It is evident that the spectrum shows bimodal intensity trend (i.e., two slopes as shown in Figure 4) which obeys the inverse power law *K f*^*−β*^ where *β* and *K* are the spectral index and spectral strength, respectively [20]. The two slopes of the PSD in this region were found to be *β*_1_ *=* 6.0 and *β*_2_ *=* 6.5. Since *β* is related to α by the equation α = (*β* − d)/2 [21], where the line scan dimension *d* is 2, then α_1_ = (6–2)/2 = 2 and α_2_ = (6.5 − 2)/2 = 2.25. Here, α_1_ attributes to the roughness exponent associated to the first FLG layer and α_2_ could be assigned to roughness exponent of the second FLG layer.

**Figure 5:**
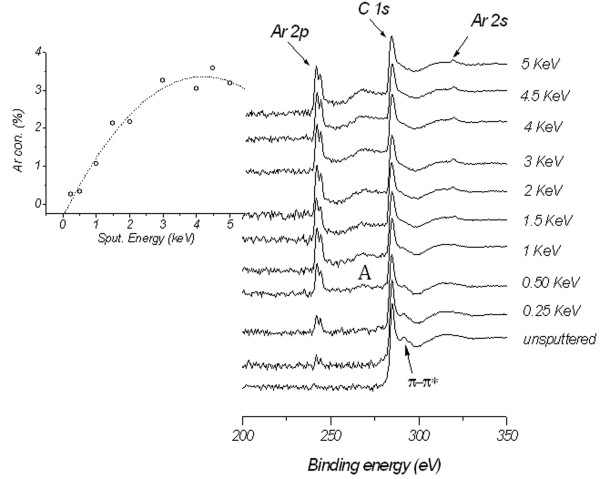
**XPS spectra after Ar**^**+**^**ion irradiation at different energies from 0.25 to 5 keV.** The spectrum for the un-irradiated sample is also plotted as a reference. Inset shows the Ar^+^ ion concentration as a function of irradiation energy.

Figure 5 shows the XPS survey spectra indicating the C (1*s*), Ar (2*p*), and Ar (2*s*) core level excitations recorded from FLG samples after Ar^+^ ion irradiation at different energies. It is clear from the XPS spectra and from the inset of Figure 5 that, as the irradiation energy increases, Ar^+^ ions are implanted into the FLG and reached the saturation limit at energies ≥ 3 keV.

Also, variation in the effect of ion irradiation can be observed from the newly developed shoulder (shoulder labeled A) in the XPS spectra along with the increasing of the irradiation energy. The origin of shoulder A is unclear at the moment; however, complex defects previously theoretically predicted [7] to be associated with Ar^+^ irradiation might be the reason. In addition, the commonly observed π − π* transition in graphene at binding energy of 290 eV [22] seems to gradually disappear as the irradiation energy is increased, indicating the FLG electronic damage. This is supported by the following further XPS investigations carried out to determine the transition from the *sp*^2^-bonding to *sp*^3^-hybridized state as a result of Ar^+^ ion irradiation.

It can be concluded that the resilience (i.e., only two layers being sputtered with Ar^+^ at 5 keV for 30 min) of FLG to the Ar^+^ ion irradiation stems from the combined effect of the Ar^+^ implantation as evident from Figure 5 and back scattering or reflection of the incoming ions due to the sample tilting. As the implanted Ar^+^ ions build up, they electrostatically repel subsequent incoming incident ions, therefore reducing number of ions involved in collisions. This charge repulsion can be understood from the ion concentration saturation observed in the inset of Figure 5 at energies ≥3 keV; a caution should be taken here as re-sputtering of the implanted Ar^+^ ions can also take place and lead to the saturation observed. Auxiliary experiments at constant irradiation energy and at different tilt sample angles were carried out to confirm the effect of the reflection of the incoming ions on the resilience of FLG. Indeed, the results (to be reported elsewhere) obtained from the ion irradiation of FLG samples at constant energy of 1 keV, and different sample tilt angles (*Φ*) from 0° to 80° show that the amount of implanted Ar^+^ ions seems to decrease as the tilt angle is increased.

**Figure 6:**
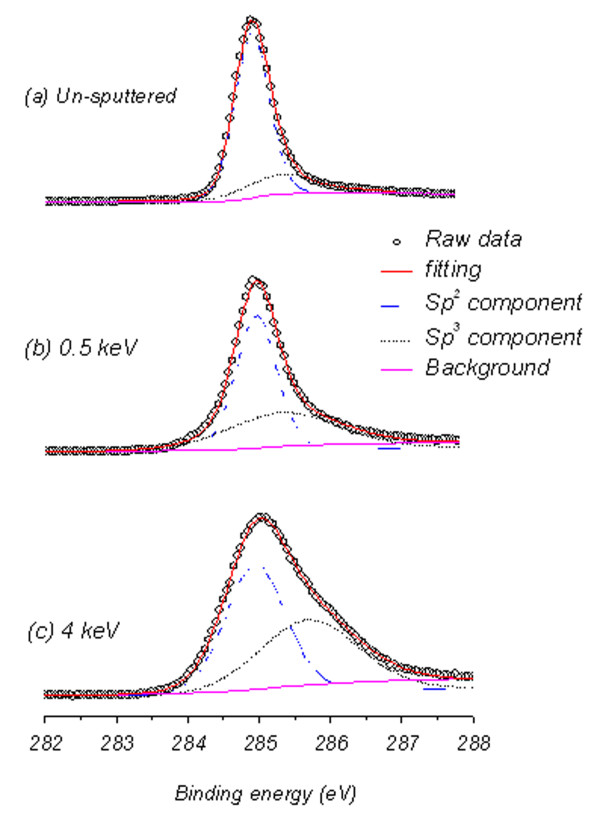
**XPS C1*s*spectra.** (**a**) Un-irradiated, (**b**) after 0.5 keV Ar^+^ ion irradiation, (**c**) after 4 keV Ar^+^ ion irradiation energy.

Figure 6 shows the fitted C (1*s*) XPS spectra of FLG samples for the un-irradiated and irradiated at energies 0.5 and 4 keV. The fitting of the spectra were done by Gaussian-Lorentzian functions with a Shirley background subtraction [23]. The fitting yields two peaks positioned at 284.8 and 285.5 eV corresponding to *sp*^2^ and *sp*^3^-hybridized states, respectively [24]. The *sp*^2^ peak appears to be shifted to higher binding energies at high Ar^+^ ion irradiation energies.

**Figure 7:**
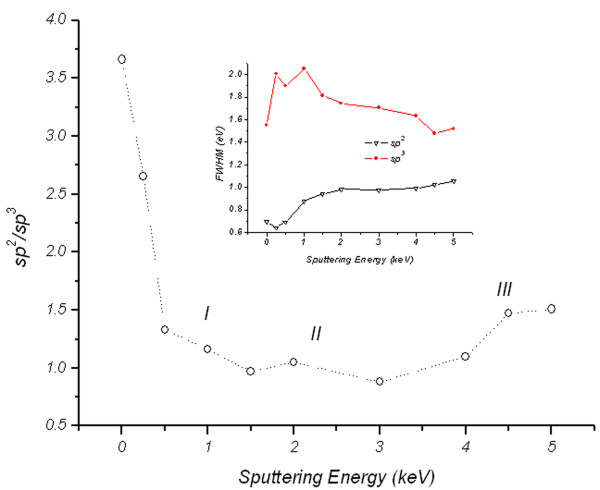
***sp***^**2**^**/*sp***^**3**^**ratio as a function of Ar**^**+**^**ion irradiation energy.** The inset shows FWHM of *sp*^2^ and *sp*^3^ components as a function of Ar^+^ ion irradiation energy.

Quantification of the *sp*^2^/*sp*^3^ ratio along with the full width at half maximum (FWHM) of the *sp*^2^ and *sp*^3^ peaks as a function of irradiation energy is shown in Figure 7 and its inset, respectively. For the un-irradiated sample, *sp*^2^ hybridization dominates (i.e., *sp*^2^/*sp*^3^ = 3.6, where *sp*^2^ = 78% and *sp*^3^ = 22%) - a FLG characteristic of a two-dimensional sheet of *sp*^2^ bonded carbon atoms in a honeycomb lattice [25-27]. However, the presence of 22% of *sp*^3^ in the un-irradiated sample is attributed either to the edge of individual graphene layers or to the intrinsic *sp*^3^ defects reported to exist in the FLG samples [28]. The well-known effect of transformation of *sp*^2^ to *sp*^3^ hybridization due to oxygen exposure of FLG [29] is ruled out in our case for the un-irradiated sample. This is justified by the absence of O 1*s* peak in the XPS spectra.

On the other hand, the initial irradiation of the FLG causes fast transformation of *sp*^2^ to *sp*^3^ hybridization as seen in region I of Figure 7. The irradiation energies in this region induce irradiation damage dominated by large number of small pits (see AFM images in Figure 1) which give less *sp*^2^ character due to the enhanced possibility of the damage of the edge of the graphene structure, consequently the formation of dangling bonds. An amorphous stable mixture containing equal *sp*^2^ and *sp*^3^ hybridizations (i.e., *sp*^2^/*sp*^3^ = 1) is observed (region II) when employing irradiation energy in the range from 1.5 to 4 keV. At high irradiation energies above 4 keV, the number of transformed *sp*^2^ bonds seems to be slightly less as depicted in region III of Figure 7 compared to those of region II. This observation can be understood in two different ways by considering the morphology formed after the irradiation in the vertical and the lateral directions. First, in the vertical direction, the observation is supported with the conclusion of the depth distribution analysis where the third graphene layer was just found to be exposed at these high irradiation energies. Therefore, XPS is expected to directly detect the *sp*^2^ contribution from this layer and the layers beneath up to approximately 2 nm of the expected XPS sampling depth in the geometry used. In the lateral direction and as seen from the AFM image at 5 keV in Figure 1, few large pits are formed due to the irradiation damage; hence, large untouched islands on the surface of the samples with better *sp*^2^ character are expected to exist. The reduced number and the large size of these pits are reflected from the long periodicity of *λ* = 72 nm and two orders of magnitude increase in PSD intensity found in Figure 3, respectively.

**Figure 8:**
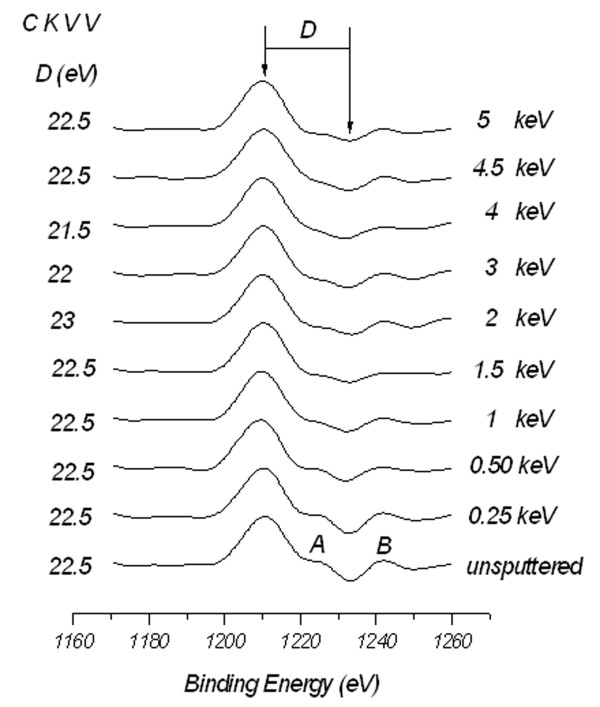
**The first derivative Auger transition C KVV spectra.** Recorded from FLG samples, as a function of Ar^+^ ion irradiation energy, indicating the values of *D* parameter.

To further account for the effect of the ion irradiation, we adapt the commonly used first derivative of C KVV spectra obtained from XPS data [30]. The selection of C KVV Auger transition reflects a self-convolution of the occupied valence band and can be used to distinguish carbon atoms at different hybridization states. Figure 8 shows the binding energy width (*D*) between the most positive maximum and most negative minimum obtained from the first derivative Auger transition C KVV spectra recorded from FLG samples, as a function of Ar^+^ ion irradiation energy.

The *D* values are within the reported values of 21 to 23 eV for HOPG (multi-layer graphene) [31], but not sensitive to the irradiation energy. However, comparison between the intensities of shoulder A and peak B reflects the *sp*^2^ to *sp*^3^ transition trend similar to that observed in Figure 7. Here, the intensity difference between A and B reaches the minimum and stabilizes after ion irradiation in energy range between 1.5 and 4 keV. Slight increase in the intensity difference is found at energies above 4 keV. Although, the shape of derivative of Auger transition C KVV spectra gives the indication of the ion irradiation effect, the *D* parameter values obtained from these spectra fail to account for *sp*^2^/*sp*^3^ hybridization transition, and reliable quantification of *sp*^2^/*sp*^3^ ratio is only obtained from the deconvolution of the XPS C (1*s*) envelope as explained before and shown in Figure 6. Our preliminary results to exploit the roughness created by ion irradiation (surfaces shown in Figure 1) for nanobubble formation and, consequently, creating graphene surfaces with less friction for different applications are promising. Supplement data shown in Figure S1 in Additional file 1 support this claim where observed contact angle, size and density of nanobubbles deposited on the sputtered samples are observed to increase with surface roughness.

From all the above findings, it is clear that more than one factor can contribute to the *sp*^2^ to *sp*^3^ transformation mechanism. For example, irradiation energy will cause lattice displacement in the FLG which affects the FLG layer cross-linking, therefore contributing in the formation of new *sp*^3^ bonds. In addition, bonds can be broken to form *sp*^3^ due to the collision impact. This is evident from the XPS reduction of π electron (π bond) intensity as the irradiation energy is increased. Furthermore, bending (to be discussed later) and breaking of the graphene planes are likely to contribute to the *sp*^2^ to *sp*^3^ transformation observed.

**Figure 9:**
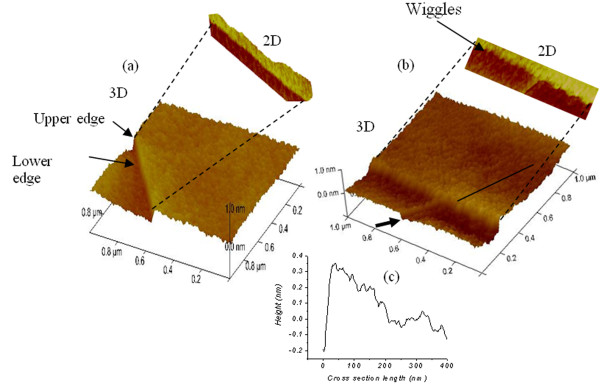
**2-D and 3-D images showing the effect of ion irradiation on the FLG edges.** (**a**) after Ar^+^ ion irradiation of 0.5 keV, (**b**) after Ar^+^ ion irradiation of 1 keV, (**c**) profile taken cross the length indicated by solid line in (b) showing shadowing effect.

We turn our discussion to the effect of Ar^+^ ion irradiation on the FLG edges. In contrary to the damage found in the flat regions, the edges of FLG are found to be much rougher; with four distinct features as shown in Figure 9, (1) the upper edge regions are not smooth and show irradiation energy dependent wiggles, and (2) the shadowing effects similar to those found in oblique angle growth of thin films are observed [32].

In this case, the edges are seen to be lifted up and shadow the regions behind them; shadowing effects are seen from the cross section profile taken at a position shown by a solid line in Figure 9b and illustrated in Figure 9c. (3) The lower edges act as weak points where more carbon sputtering takes place, and (4) new features (shown by arrow in Figure 9b) at an angle of 53° from the edges are formed as the irradiation energy is increased. All irradiation features and their roughness amplitudes after 3 keV ion irradiation can be seen in the supplement data shown in Figure S2a,b in Additional file 2. The presence of these features is detected by the fast Fourier transform (FFT) analysis shown in the inset of Figure S2b in Additional file 2.

**Figure 10:**
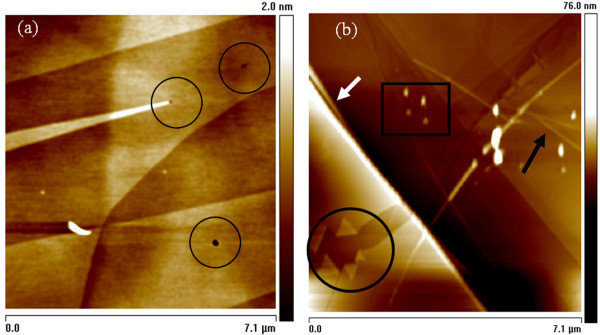
**2-D AFM images showing localized hole defects and shape of FLG folding.** (**a**) The localized hole defects after Ar^+^ ion irradiation of 1.5 keV. (**b**) Triangle shape of FLG folding (enclosed by a circle), multi-layer folding (indicated by white arrow), wrinkles (indicated by black arrow) and isolated particles (enclosed by a rectangle) after 4.5 keV ion irradiation.

During the ion irradiation, unusual defects and edge reconstructions were also observed; defects appear only after ion irradiation and were not found in the un-irradiated samples. Furthermore, these defects and edge reconstructions are similar to those reported by Zhang and Feng [33] during the growth of graphene and by Mathew et al. [34] after MeV proton beam irradiation of graphene, respectively. Figure 10a shows the AFM image of the obtained FLG after Ar^+^ ion irradiation of 1.5 keV. Few holes (enclosed by circles) can be clearly seen, suggesting the possibility of creating nanoporous graphene layers by ion irradiation for filtration applications. Increasing the ion irradiation to 4.5 keV, the peeling of graphene layers in the direction normal to the edge takes place which is associated with triangular-shaped FLG folding (enclosed by a circle) as shown in Figure 10b. Furthermore, FLG folding along the edge (indicated by white arrow) is seen to take place.

This type of folding has been observed on the surface of FLG resembling single wall nanotubes or double wall nanotubes due to the single- or double-graphene layer folding back to itself [35]. The folding observed might be due to the electron beam damage associated with scanning electron microscope and transmission electron microscope used while characterizing the samples in these studies. In our case, the folding is rather attributed to the irradiation process-induced damage which includes multi-layers of graphene as inferred from the thickness of folded layers ranging from 2 to 4 nm. Intense irradiation of the multi-folded layers can induce isolated particles (enclosed by a rectangle) and initiate wrinkles (indicated by black arrow) as seen in Figure 10b. Similar edge reconstructions have been reported by Huang et al. [36], which are due to the graphene layer-by-layer peeling as a result of void formations and their migration towards the graphene edges. This process was associated with atomic sublimation caused by Joule heating and facilitated by atomic displacement caused by high-energy electron irradiation. Despite the serious electronic and morphological damages at high energies, roughness, PSD, XPS, π − π* transition, *sp*^2^/*sp*^3^ ratio, *sp*^2^ and *sp*^3^ FWHM values and Auger transition C KVV spectra data analysis reveal that, using low energy of 0.25 keV Ar^+^ ion irradiation at large sample tilt angle at room temperature, is an alternative way of peeling graphene layers without the need of Joule heating.

## Conclusion

Ar^+^ ion irradiation of FLG has been investigated in which ripple structure formation with varying amplitude and periodicity predominantly in the first two graphene layers was observed. In addition, *sp*^2^ to *sp*^3^ bonding transformation through the quantification of the deconvolution of the XPS C (1*s*) envelope along with electronic damage as deduced from the gradual disappearance of the π − π* transition in graphene, as a function of irradiation energy, was found. Although the shape of derivative of Auger transition C KVV spectra provided an indication of the ion irradiation effect, the *D* parameter values obtained from these spectra failed to account for *sp*^2^ to *sp*^3^ hybridization transition. Among others, energy-dependent edge wiggles, shadowing effects, defects (such as holes and FLG edge reconstructions) and edge folding which depend on the sputtering conditions were all found. The existence of these defects suggests the possibility of creating nanoporous FLG by ion irradiation and the development of high density nanobubble FLG surfaces for various applications. Furthermore, the combined effect of Ar^+^ implantation and sample tilt angle on FLG irradiation can explain the ion irradiation resilience of FLG. Although we looked specifically at Ar^+^ irradiation, our results can also provide insights into the response of FLG to irradiation by other noble gasses or other species. Despite the mixing of *sp*^2^ and *sp*^3^ is not desirable for the electronic properties of FLG, coming up with applications which depend on tuning *sp*^2^/*sp*^3^ ratio will be essential to fully appreciate the importance of the ion irradiation of FLG. In addition, future investigations at atomic scale for the edge reconstructions and other defects will be essential to support the results of this study.

## Competing interests

The authors declare that they have no competing interests.

## Authors’ contributions

SHA contributed in the overall project design and implementation. ME and MA contributed in the data analysis. AK contributed in the atomic force microscopy experiments. TH contributed in the few layer graphene sample preparation and sputtering. MTZM contributed in the X-ray photoemission spectroscopy experiment and manuscript preparation. MRA contributed in the manuscript preparation. All authors read and approved the final manuscript.

## Authors’ information

SHA is an associate professor in Nanotechnology and Surface Science. ME is a professor in Theoretical Physics, doing first principle calculations. MA is an associate professor in Theoretical Physics and Computational Simulation Physics. AK is a masters degree student. TH is a Ph.D. student working on swift ions in materials. MTZM is a Ph.D. student working on nanotechnology. MRA is a professor in magnetism and head of the department.

## Supplementary Material

Additional file 1 **Effect of surface roughness on the nanobubble formation.** Figure S1a,b show 2-D and 3-D AFM images of nanobubbles formed on the unsputtered FLG sample. The height and lateral size of the bubbles range from 2 to 5 nm and from 15 to 30 nm, respectively. It is also clear that the bubbles are well dispersed in a random fashion. However, agglomeration and an increase in their size and number density are prominent features of the bubbles grown on the irradiated FLG samples as seen from Figure S1(c, d) and (e, f), respectively. Click here for file

Additional file 2 **Irradiation features and roughness amplitudes after 3-keV ion irradiation.** Figure S2. (a) 3-D image showing the effect of ion irradiation on the FLG edges after Ar^+^ ion irradiation of 3 keV. A, B and C denote the irradiated edges, cross sputtered areas at 53° from A direction and shadowing features, respectively. (b) PSD obtained from the image shown in (a) showing the amplitude of the surface's roughness of features A, B, and C as a function of the spatial frequency. FFT of the image shown in (a) revealing the presence of features A, B and C is shown in the inset of (b). Click here for file
