# Supplementary material for: Unusual surface and edge morphologies, sp2 to sp3 hybridized transformation and electronic damage after Ar+ ion irradiation of few-layer graphene surfaces
Source: Nanoscale Res Lett. 2012 Aug 19;7(1):466. doi: 10.1186/1556-276X-7-466 (PMC3496642; doi:10.1186/1556-276X-7-466)
Supplement: Additional file 1 — Effect of surface roughness on the nanobubble formation. Figure S1a,b show 2-D and 3-D AFM images of nanobubbles formed on the unsputtered FLG sample. The height and lateral size of the bubbles range from 2 to 5 nm and from 15 to 30 nm, respectively. It is also clear that the bubbles are well dispersed in a random fashion. However, agglomeration and an increase in their size and number density are prominent features of the bubbles grown on the irradiated FLG samples as seen from Figure S1(c, d) and (e, f), respectively. [file 1556-276X-7-466-S1.doc]

**Effect of surface roughness on the nanobubbles formation**

Figures S1(a) and (b) show 2D and 3D AFM images of nanobubbles formed on the un-sputtered FLG sample. The height and lateral size of the bubbles ranges from 2 nm to 5 nm and from 15 nm to 30 nm respectively. It is also clear that the bubbles are well dispersed in a random fashion. However, agglomeration and an increase in their size and number density are prominent features of the bubbles grown on the irradiated FLG samples as seen from figures S1(c, d) and (e, f) respectively.


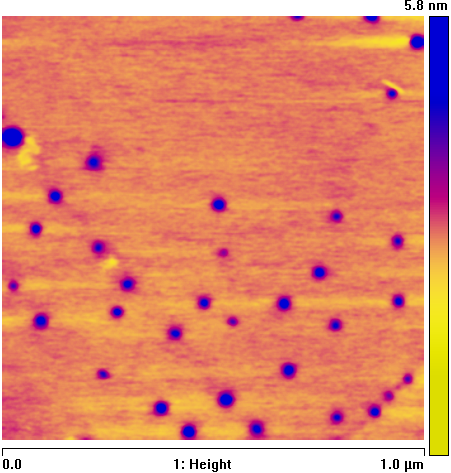

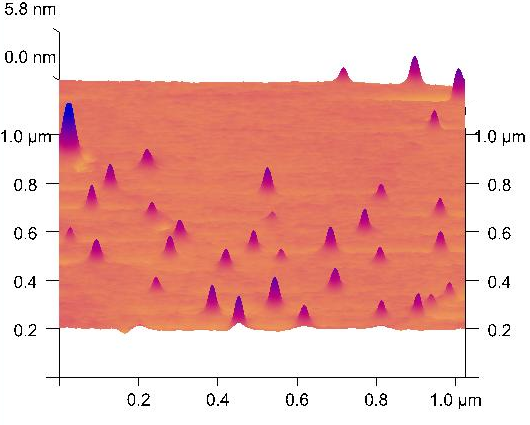

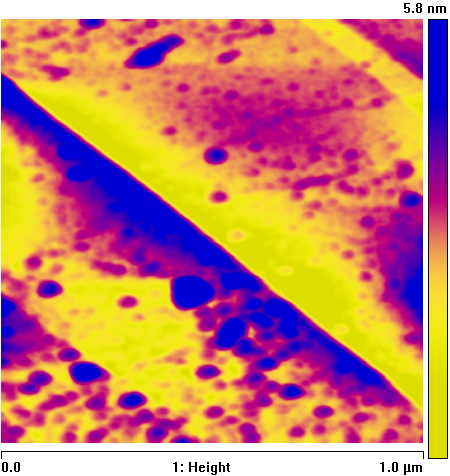

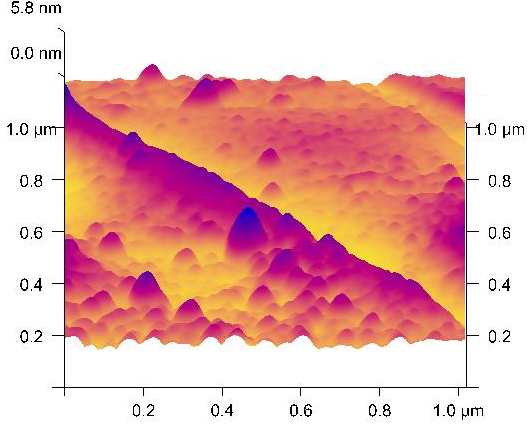

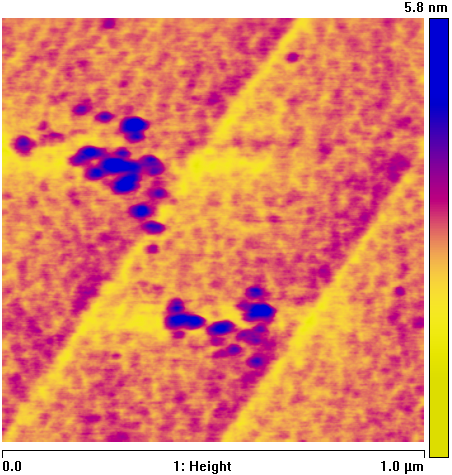

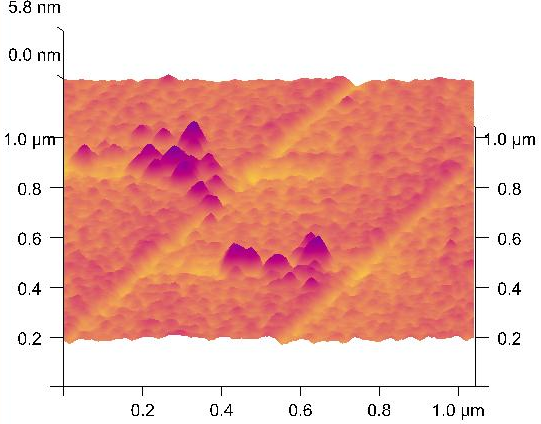


(a)

(a)

(b)

(c)

(d)

(e)

(f)

Figure S1: (a) and (b) are 2D and 3D AFM images showing nanobubbles formation on the un-sputtered sample respectively, (c) and (d) are 2D and 3D AFM images showing nanobubbles clustering on the irradiated sample after Ar^+^ ion irradiation of 1.5 keV respectively, (e) and (f) are 2D and 3D AFM images showing an increase in the nanobubble density after ion irradiation of 4 keV respectively.

It has been suggested that concave areas in rough surfaces are unfavorable for water penetration, hence allowing these areas to act as gas cavities to form low curvature nanobubbles with enhanced stability. Also it has been reported that rough surfaces do provide large sites for nanobubbles formation, therefore justifying their population increase and their agglomeration on rough surfaces. The above explanation coupled with the fact that energy irradiation (i.e. increase in roughness) is expected to increase FLG hydrophobicity fits very well with the observation in this work of an increase of nanobubbles density and contact observed angles, agglomeration and nanobubbles size reduction.
