# Supplementary material for: Unusual surface and edge morphologies, sp2 to sp3 hybridized transformation and electronic damage after Ar+ ion irradiation of few-layer graphene surfaces
Source: Nanoscale Res Lett. 2012 Aug 19;7(1):466. doi: 10.1186/1556-276X-7-466 (PMC3496642; doi:10.1186/1556-276X-7-466)
Supplement: Additional file 2 — Irradiation features and roughness amplitudes after 3-keV ion irradiation. Figure S2. (a) 3-D image showing the effect of ion irradiation on the FLG edges after Ar+ ion irradiation of 3 keV. A, B and C denote the irradiated edges, cross sputtered areas at 53° from A direction and shadowing features, respectively. (b) PSD obtained from the image shown in (a) showing the amplitude of the surface's roughness of features A, B, and C as a function of the spatial frequency. FFT of the image shown in (a) revealing the presence of features A, B and C is shown in the inset of (b). [file 1556-276X-7-466-S2.doc]

###

(b)


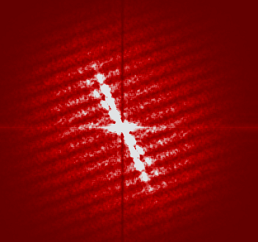

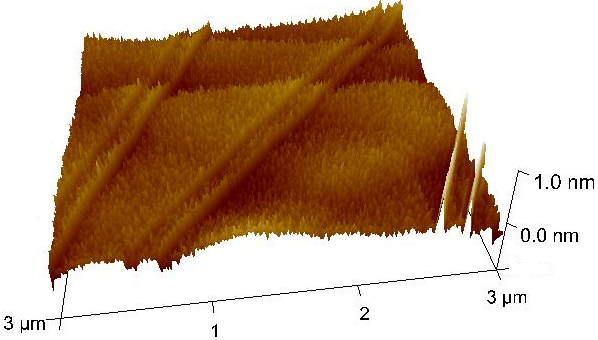


B

B

A

A

A

C

C

C

53°

(a)

Figure S2: (a) 3D image showing the effect of ion irradiation on the FLG edges after Ar^+^ ion irradiation of 3 keV: A, B and C denote the irradiated edges, cross sputtered areas at 53° from A direction and shadowing features respectively, (b) PSD obtained from the image shown in (a) showing the amplitude of the surface’s roughness of features A, B, and C as a function of the spatial frequency. FFT of the image shown in (a) revealing the presence of features A, B and C is shown in the inset of (b).
